# Perceived Challenges to Routine Uptake of the Ankle Brachial Index within Primary Care Practice

**DOI:** 10.3390/jcm10194371

**Published:** 2021-09-24

**Authors:** Lily Y. C. Chiu, Muzammil H. Syed, Abdelrahman Zamzam, Ori D. Rotstein, Rawand Abdin, Nadine Laraya, Mohammad Qadura

**Affiliations:** 1 Division of Vascular Surgery, St. Michael’s Hospital, Toronto, ON M5B 1W8, Canada; lily1103@yorku.ca (L.Y.C.C.); muzammil.syed@mail.utoronto.ca (M.H.S.); abdelrahman.zamzam@unityhealth.to (A.Z.); 2 Department of Surgery, University of Toronto, Toronto, ON M5S 1A1, Canada; ori.rotstein@unityhealth.to; 3 Keenan Research Centre for Biomedical Science, Li Ka Shing Knowledge Institute of St. Michael’s Hospital, Toronto, ON M5B 1W8, Canada; 4 Department of Medicine, McMaster University, Hamilton, ON L8S 4K1, Canada; rawand.abdin@medportal.ca; 5 Family Medicine Department, St. Joseph’s Health Centre, Toronto, ON M6R 1B5, Canada; nadine.laraya@unityhealth.to; 6 Department of Family and Community Medicine, Temerty Faculty of Medicine, University of Toronto, Toronto, ON M5G 1V7, Canada

**Keywords:** peripheral arterial disease (PAD), ankle-brachial pressure index (ABI), primary care practitioners (PCP)

## Abstract

(1) Introduction: The ankle-brachial index (ABI) is the most widely used method of diagnosing peripheral arterial disease (PAD). However, the uptake of ABIs has been reported to be low in primary care settings across different various healthcare settings; however, this is yet to be investigated within the Canadian context. (2) Objective: Therefore, we sought to assess the rates of ABI usage as well as perceived barriers among primary care practitioners (PCPs) in Toronto, Canada. (3) Methods: A modified questionnaire was electronically sent to 257 PCPs in the Greater Toronto Area (GTA). Questions pertained to frequency, feasibility, utility, and barriers associated with ABI usage in clinical practice. Responses were collected and tallied. (4) Results: A total of 52 PCPs completed the questionnaire. 79% of PCPs did not routinely perform ABIs within their clinical practice, and 56% deemed ABI usage as unfeasible. Constraints in time and staff personnel, as well as complexity of ABI result interpretation, were cited as the major perceived barriers to ABI usage. The overwhelming majority of PCPs viewed alternative forms of diagnosis, such as a blood test for PAD, as being preferable to ABI, as such an approach would enhance diagnostic simplicity and efficiency. (5) Conclusion: ABI usage rates are poor within primary care practices in Toronto, Canada. Alternative approaches for diagnosing PAD may result in greater adoption rates among PCPs and therefore improve the identification of patients with PAD.

## 1. Introduction

Peripheral arterial disease (PAD) is a chronic atherosclerotic disease characterized by the narrowing or complete blockage of the blood vessels to limbs [1,2]. Over 200 million individuals around the world are affected by PAD, with adults in low to middle income countries (140 million) more disproportionately affected than individuals in Europe (40.5 million), North America (14.3 million), and other western high-income countries (6.5 million) [2,3]. Furthermore, due to the gaining of the population, the incidence rate of PAD is rapidly increasing each year [4].

Symptomatic patients with PAD normally present with intermittent claudication during ambulation, and with lower limb numbness, pain at rest, or tissue loss in advanced cases of PAD, known as chronic limb threatening ischemia (CLTI). Interestingly, the vast majority of patients with PAD are asymptomatic, which greatly complicates diagnosis for primary care practitioners (PCPs) [3,4,5]. If left undiagnosed and untreated, PAD can result in detrimental consequences, such as lower-limb amputations and mortality [4,6].

Currently, the ankle-brachial pressure index (ABI) is the most widely used method of PAD diagnosis among PCPs [1]. The ABI takes the higher of the two systolic blood pressures of the anterior tibial artery or posterior tibial artery and divides it by the average of the right and left brachial artery pressures [7]. An ABI of <0.9 is indicative of the presence of PAD [8]. In rare cases, patients with PAD that have a calcified tibial vessel may present with ABI values exceeding 1.4 [2,4].

According to the PAD guidelines developed by the American Heart Association, patients that meet one or more of the following criteria require routine screening of PAD: (a) age <50 years, with diabetes and one other atherosclerosis risk factor, (b) age 50–69 with a history of smoking or diabetes, (c) age ≥70 years, (d) symptoms of claudication or ischemic rest pain, (e) abnormal lower extremity pulse examination, and/or (f) known atherosclerotic disease [9]. With regards to asymptomatic patients, guidelines and suggestions require more clarification [4].

The ABI has many advantages. For instance, it is non-invasive and relatively simple to perform [8]. Despite these advantages, the uptake of ABI remains low within general practice [10]. Studies indicate that up to 50% of PCPs cite limitations in clinical settings as being a barrier to ABI usage [11], and up to 42% of PCPs perform ABIs out of compliance with guidelines [10]. As a result, ABI is underutilized and measurements often yield inconsistent results among different PCPs [7]. Therefore, it comes as no surprise to see a growing body of recent scientific literature calling for improved education, training and resources targeted towards PCPs for PAD diagnosis [10], as well as clearer guidelines on ABI usage [7,10].

Currently, Ontarians with a stable PAD presenting to PCPs ought to be managed at the PCP office via counselling, exercise, and medications (anti-platelet, hypoglycemic and or anti-hypertension medication). Referrals to vascular specialists are recommended in cases when there are: (a) lifestyle factors hindering disease management, (b) resistance to medicinal intervention, and/or (c) advanced disease course requiring surgical intervention. In cases where ABI testing is unfeasible in PCP offices, either due to non-compressible ABI or wound presence, PCPs have the option of referring patients to vascular laboratories. However, vascular laboratories in Ontario are relatively limited in number, and most have long wait-list times associated with them.

PCPs trained and educated in the correct usage of ABI remark on its effectiveness and importance in diagnosing PAD [1]; however, data within the Canadian context is limited in this regard. Therefore, the objective of this study was to assess the rate of ABI usage and their perceived barriers to usage among PCPs practicing in a large urban setting.

## 2. Materials and Methods

### 2.1. Questionnaire Distribution

A cross-sectional survey was conducted, using a modified questionnaire from a previously conducted UK study [1]. Minor adaptations and additions were made to the questionnaire as per our study aims and objectives. After obtaining permission for adaptation and re-use, an electronic version of the questionnaire was sent by the chair of a large PCP community to 257 PCPs located in the Greater Toronto Area (GTA). Two additional reminders were sent out to promote completion of the questionnaire. With a population of ~7.0 million, the GTA accounts for 48% of Ontario’s population [12].

A total of 10 questions comprised the questionnaire, of which the vast majority were close-ended, multiple-choice questions (Figure S1). The first question assessed the knowledge of the respondents regarding available diagnostic modalities and risk factors for PAD. The following questions focused on the utility of ABI as a diagnostic test. These included questions pertaining to the frequency of ABI usage in their primary care practice, the personnel performing the ABI, as well as the feasibility, utility, and barriers associated with the ABI. Finally, participants were asked to give their opinion on a potential introduction of a diagnostic blood test for PAD. All respondents of the questionnaire were anonymous.

### 2.2. Statistical Analysis

Descriptive data were expressed as means with standard deviations (SD), or frequencies with percentages, as appropriate. For missing survey data, whether total non-response or partial response, a list-wise deletion method was used, in which any participant with a missing value on a certain variable was deleted from the analysis of that specific variable. SPSS software, version 23 (SPSS Inc., Chicago, IL, USA) was used for data entry and statistical analysis, whereas Microsoft Excel was used for graphical illustrations.

### 2.3. Research Ethics

Ethics approval was obtained from the research ethics board at St. Michael’s Hospital, Unity Health Toronto on October 19, 2020 (REB# 19-341). All aspects of the study were conducted according to the guidelines of the Declaration of Helsinki, and all respondents consented to participate in this study.

## 3. Results

A total of 52 PCPs with patients with PAD completed the survey (response rate of 20.2%).

### 3.1. Perceived Barriers, Limitations, and Feasibility of ABI Usage

The top three limiting factors/perceived barriers to ABI usage among PCPs were time constraints (77%), office staff availability (65%) and their confidence in ABI interpretation (60%) (see Table 1). Additionally, the vast majority of respondents deemed the performance of ABI procedure in the office setting to be unfeasible (56%), with the remainder considering the ABI somewhat feasible (23%) or very feasible (21%).

### 3.2. Use of ABI in Primary Care Practice

**Figure 1:**
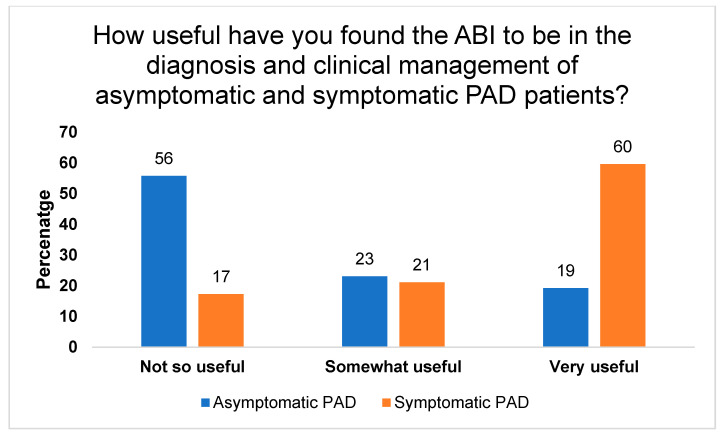
Distribution of responses of 52 GPs to the question “How useful have you found ABI to be in the diagnosis and clinical management of asymptomatic and symptomatic PAD patients?”.

Our data showed that 79% of PCPs indicated that the ABI is not routinely performed in their practice (*n* = 41). According to the respondents, 44% have never previously used the ABI, 39% perform ABI on patients annually, 12% conduct the ABI monthly, and only 4% perform the ABI on a weekly basis. Unsurprisingly, the majority of PCPs found the ABI to be more useful in the diagnosis and clinical management of symptomatic patients (60%) as compared with asymptomatic patients (19%) (see Figure 1). Lastly, the respondents indicated that the ABI would be most useful to screen for PAD in patients with diabetes (83%), advanced age (60%) and chronic renal failure (50%) (refer to Table 2).

### 3.3. Factors Important for Diagnosing PAD

Most of the respondents agreed that risk factors (92%), ABI (87%) and pulse examination (85%) are important in pointing to a diagnosis of PAD (see Table 3). This highlights that PCPs have received training and education on how to screen patients for PAD.

### 3.4. Other Potential Diagnostic Methods/Tools

Lastly, the respondents were quizzed on their openness to implementing new diagnostic tools for PAD in their clinical practice, such as a clinically validated blood test. An overwhelming majority of PCPs (92%) indicated their interest in such a test, and 83% of respondents expressed their desire to incorporate a PAD blood test routinely in their clinical practice for screening purposes. Furthermore, 58% of PCPs would prefer this blood test to take the form of a point of care test, given their efficiency (diagnosis time < 15 min) and simplicity.

## 4. Discussion

In this study, we sought to assess the rate of usage and perceived barriers of ABI usage among PCPs in Toronto. Our data suggest that the majority of PCPs who responded: (1) do not routinely perform the ABIs in their clinical practice, considering it unfeasible and fraught with a number of barriers and limiting factors, and (2) would be more open to incorporating more efficient and simpler innovations for PAD-related diagnosis/screening, such as a point-of-care blood test.

Our results complement findings described in other studies. A study by Yap Kannan R et al. assessing ABI use and perceived barriers among general practitioners in the UK identified similar rates of certain limiting factors, such as time constraints (84% vs. 77% in our study) and staff availability (89% vs. 65% in our study) [1]. In contrast, the complexity of ABI interpretation was a major perceived barrier in our study (60%), but not in the study conducted by Yap Kanan R. et al. [1] Difficulty in interpreting ABI results may be attributed to the varying practices on ABI usage [9,10,13], or a lack of training in ABI usage and interpretation [1,10,12,13]. For instance, previous data suggests that an intra-variability of 0.005 is yielded in ABI readings over 6 different days [11]. Inter-variability for ABI testing is expected to be even higher. The variance in ABI measurements, and the associated difficulty of interpreting it, may explain why only 44% of PCPs in our study found the ABI to be feasible, in comparison to 69% in Yap Kannan R. et al.’s study [1]. It may also explain why 79% of PCPs in our study do not routinely perform ABI in their clinical practice—a significantly higher proportion than that observed in the studies by Yap Kannan R et al. (~53%) [1] and Davies et al. (22%) [10], among others [11]. However, considered together, all these studies suggest a limited usage of ABIs within primary care in different healthcare settings, in spite of its proven secondary role in protecting against cardiovascular-related events and mortality [14].

Despite the variability of ABI use across varying healthcare systems, there is a common understanding among PCPs that early diagnosis of PAD is extremely important [1,2,6]. Respondents from our study expressed enthusiasm at the prospect of an alternative approach to diagnosis though incorporating a novel diagnostic blood test for PAD. While potential blood-based biomarkers for PAD have been previously identified, none of them are currently commercially available [13,14,15,16,17,18,19]. Furthermore, while other diagnostic modalities are available, they are yet to be widely adopted [1]. For instance, technologies such as the Photoplethysmography (PPG), previously reported in the British Journal of General Practice in 2015, is still experiencing adoption limitations [5]. Therefore, there is a gap that calls for new tools and technologies for screening PAD. In order for such technology/tools to acquire a successful clinical adoption, it must be accurate, affordable, efficient, and simple to perform. This study has the following limitations: Firstly, the survey had a relatively small respondent rate (20%); however, we still managed to get a respectable sample size (*n* = 52). Secondly, focus groups and interviews were not conducted as part of this study, which potentially could have yielded some great insights and findings. Lastly, participants of this survey were limited to the GTA, which may not reflect the opinions of PCPs nationally across Canada.

## 5. Conclusions

In conclusion, our findings suggest that PCPs in the GTA do not routinely perform the ABI in their clinical practice due to constraints on time and personnel, as well as difficulties in interpreting the ABI results. Alternate modalities of diagnosing PAD that are simpler and more efficient in diagnosing PAD may increase uptake and adoption of PAD screening among PCPs.

## Figures and Tables

**Table 1:** Results to the question, “What factors, if any, limit the utilization of the ABI in your clinical practice?” (*n* = 52).

Limitations	Major Limitation *n* (%)	Minor Limitation *n* (%)	No Limitation *n* (%)
Time Constraint	40 (77%)	8 (15%)	4 (8%)
Financial Constraint	18 (35%)	15 (29%)	19 (36%)
Clinical Significance	12 (23%)	14 (27%)	24 (46%)
Staff Availability	34 (65%)	13 (25%)	4 (8%)
Patient Willingness	8 (15%)	11 (21%)	33 (64%)
Presence of Wounds	16 (31%)	15 (29%)	20 (39%)
ABI Interpretation	31 (60%)	11 (21%)	10 (19%)

**Table 2:** Distribution of results to the question, “Which of the following patient population is the ABI considered a good PAD screening tool in your opinion?” (*n* = 52).

Patient Population	Yes *n* (%)	No *n* (%)
Healthy Patients	5 (10%)	47 (90%)
Diabetics	43 (83%)	9 (17%)
Chronic Renal Failure	26 (50%)	26 (50%)
Elderly (>65 Years old)	31 (60%)	21 (40%)

**Table 3:** Results for question, “Which of the following do you consider important in diagnosing PAD?” (*n* = 52).

Factors	Agree *n* (%)	Disagree *n* (%)
Risk Factors	48 (92%)	4 (8%)
Pulse Examination	44 (85%)	8 (15%)
Questionnaires	8 (15%)	44 (85%)
Ankle-Brachial Index (ABI)	45 (87%)	7 (13%)
Toe Brachial Index (TBI)	6 (12%)	46 (88%)

## Data Availability

All relevant data can be found in this manuscript.

## Supplementary Materials

The following are available online at https://www.mdpi.com/article/10.3390/jcm10194371/s1, Figure S1. Peripheral Arterial Disease Survey Administered to PCPs in Toronto, Canada.
