# Supplementary material for: Perceived Challenges to Routine Uptake of the Ankle Brachial Index within Primary Care Practice
Source: J Clin Med. 2021 Sep 24;10(19):4371. doi: 10.3390/jcm10194371 (PMC8509610; doi:10.3390/jcm10194371)
Supplement: Supplementary file 1 [file jcm-10-04371-s001.zip › jcm-1338663-supplementary.pdf]

Reproduced and adapted from [Survey of ankle–brachial pressure index use and its perceived barriers by general practitioners in the UK, Kannan RY, Dattani N, Sayers RD, Bown MJ, 92(1088), 322–327, 2021] with permission from BMJ Publishing Group Ltd.

Peripheral Arterial Disease Screening Survey

Please fill this questionnaire which assesses current practice in the diagnosis of peripheral arterial disease (PAD) at primary care facilities.

1. Which of the following do you consider important in diagnosing PAD?

Risk Factors	☐ Yes | ☐ No | ☐ Unsure
Pulse Examination	☐ Yes | ☐ No | ☐ Unsure
Questionnaires	☐ Yes | ☐ No | ☐ Unsure
Ankle Brachial Index (ABI)	☐ Yes | ☐ No | ☐ Unsure
Ankle Pressure	☐ Yes | ☐ No | ☐ Unsure
Toe Brachial Index (TBI)	☐ Yes | ☐ No | ☐ Unsure
Toe Pressure	☐ Yes | ☐ No | ☐ Unsure

2. Who performs the ABI at your practice?

☐ General Practitioner ☐ Registered Nurse

☐ Nurse Practitioner ☐ Healthcare Assistant

☐ Technician ☐ Not routinely performed in my practice

3. How often do you use the ABI?

☐ Weekly ☐ Annually

☐ Monthly ☐ Never

4. How useful have you found the ABI to be in the diagnosis and clinical management of

Asymptomatic PAD:

☐ 1 ☐ 2 ☐ 3 ☐ 4 ☐ 5

Not Useful — Very Useful

Symptomatic PAD:

☐ 1 ☐ 2 ☐ 3 ☐ 4 ☐ 5

Disappointing — Exceptional

Presence of Wounds:

☐ 1 ☐ 2 ☐ 3 ☐ 4 ☐ 5

No limitation — Major limitation

Performing Handheld:

☐ 1 ☐ 2 ☐ 3 ☐ 4 ☐ 5

No limitation — Major limitation

Doppler Examination:

☐ 1 ☐ 2 ☐ 3 ☐ 4 ☐ 5

No limitation — Major limitation

ABI Interpretation:

☐ 1 ☐ 2 ☐ 3 ☐ 4 ☐ 5

No limitation — Major limitation

5. How feasible is incorporating the ABI into your daily practice?

☐ 1 ☐ 2 ☐ 3 ☐ 4 ☐ 5

Not feasible — Very feasible

6. Is ABI a good screening tool for PAD in the following patients?

Healthy patients	☐ Yes | ☐ No | ☐ Unsure
Diabetics	☐ Yes | ☐ No | ☐ Unsure
Chronic renal failure	☐ Yes | ☐ No | ☐ Unsure
Elderly (>65 years)	☐ Yes | ☐ No | ☐ Unsure

7. What factors if any, limit the utilization of the ABI in your clinical practice?

Time Constraints:

☐ 1 ☐ 2 ☐ 3 ☐ 4 ☐ 5

No limitation — Major limitation

Financial Constraints:

☐ 1 ☐ 2 ☐ 3 ☐ 4 ☐ 5

No limitation — Major limitation

Clinical Significance:

☐ 1 ☐ 2 ☐ 3 ☐ 4 ☐ 5

No limitation — Major limitation

Staff Availability:

☐ 1 ☐ 2 ☐ 3 ☐ 4 ☐ 5

No limitation — Major limitation

Patient Willingness:

☐ 1 ☐ 2 ☐ 3 ☐ 4 ☐ 5

No limitation | Major limitation

Staff Training:

☐ 1 ☐ 2 ☐ 3 ☐ 4 ☐ 5

No limitation | Major limitation

Availability of Doppler:

☐ 1 ☐ 2 ☐ 3 ☐ 4 ☐ 5

No limitation | Major limitation

Availability of Cuffs:

☐ 1 ☐ 2 ☐ 3 ☐ 4 ☐ 5

No limitation | Major limitation

Application of Cuff:

☐ 1 ☐ 2 ☐ 3 ☐ 4 ☐ 5

No limitation | Major limitation

8. If a diagnostic **blood test** (with similar diagnostic accuracy as ABI) for PAD becomes available, would you order it (replaces the need for ABI)?

☐ Yes | ☐ No | ☐ Unsure

9. Would you incorporate this PAD-blood test into your clinical practice to screen for PAD?

☐ Yes | ☐ No | ☐ Unsure

10. Would you prefer this blood test to take the form of a point of care test that can give you a diagnosis within 15 minutes?

☐ Yes | ☐ No | ☐ Unsure

Figure S1. Peripheral Arterial Disease Survey Administered to PCPs in Toronto, Canada.
